# Study on the hypolipidemic effect of *Inonotus obliquus* polysaccharide in hyperlipidemia rats based on the regulation of intestinal flora

**DOI:** 10.1002/fsn3.3052

**Published:** 2022-09-25

**Authors:** Fengyuan Lin, Xiao Li, Xiao Guo, Xuechun Lu, Xiao Han, GuangYu Xu, Peige Du, Liping An

**Affiliations:** ^1^ College of Pharmacy Beihua University Jilin China; ^2^ General Hospital of the People's Liberation Army Beijing China

**Keywords:** hyperlipidemia, *Inonotus obliquus*, intestinal flora, polysaccharide

## Abstract

The purpose of this study was to observe the effect of *Inonotus obliquus* polysaccharide (IOP) on blood lipids and its regulation on the intestinal flora in hyperlipidemia rats, and explore the modern biological connotation of IOP in reducing blood lipids. In this study, we obtained the crude IOP by the water extraction and alcohol precipitation method, and then classified it by DEAE ion‐exchange chromatography to obtain the acidic *I. obliquus* polysaccharide (IOP‐A). After the administration of the IOP‐A, the serum TC, TG, and LDL‐C levels were significantly lower, while the serum HDL‐C levels were significantly higher. The expression of CYP7A1 protein was considerably increased, whereas the expression of SREBP‐1C protein was considerably decreased in the rat hepatic tissue. In addition, the IOP‐A could significantly alleviate the hepatocyte fatty degeneration in the liver lobule of rats. We believe that the IOP‐A can affect the composition of intestinal flora by reducing the relative abundance of Firmicutes and increasing the relative abundance of Bacteroidetes. These findings indicated that the IOP‐A can regulate the dyslipidemia of hyperlipidemia rats, and its mechanism may be through regulating the CYP7A1 and SREBP‐1C expression in the metabolism of lipids, and correcting the imbalance of intestinal flora structure caused by a high‐fat diet.

## Graphical abstract

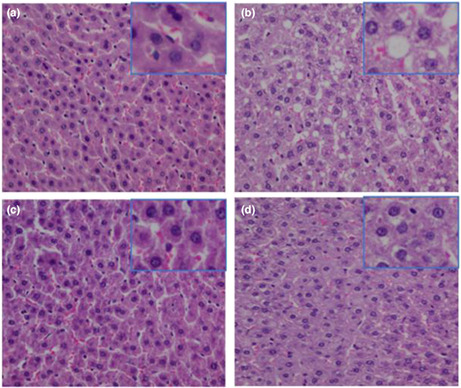

## 1 INTRODUCTION

Hyperlipidemia is a common dyslipidemia disease that can lead to fatty liver, and some cardiovascular and cerebrovascular diseases, such as atherosclerosis and coronary heart diseases, seriously endangering human health (Woo et al., 2018). The occurrence of hyperlipidemia is due to the abnormality of fat metabolism or transport, which makes one or more lipids in serum higher than the normal range, as evidenced by an increase in the serum total cholesterol (TC), triglyceride (TG), and low‐density lipoprotein cholesterol (LDL‐C), as well as a decrease in high‐density lipoprotein cholesterol (HDL‐C) (Lv et al., 2019). With the influence of many factors such as people's eating habits, the incidence of hyperlipidemia and related diseases have been increasing year by year now.

*Inonotus obliquus*, also known as birch mushroom, chaga, *Tricholoma obliquus* and *Ganoderma sibirica*, belongs to *Poria hypobrunnea Petch*, *Polyporaceae*, *Polyporales*, *Hymenomycetes*, *Basidiomycotina*, *Eumycota*, and it parasitizes on the damaged parts of trunk or bark of birch, elm and red poplar, and is one of the world's top 10 precious medicinal fungi (Liu et al., 2015). *I. obliquus* contains more than 200 bioactive substances, such as terpenoids, steroids, corticosteroids, melanin, polyphenols, and polysaccharides (Jiang et al., 2017). *I. obliquus* polysaccharide (IOP) is the main active substance of *I. obliquus*, with the physiological functions of anti‐cancer, anti‐inflammation, anti‐oxidation, hypoglycemia, and enhancing immunity (Ding et al., 2017; Xia et al., 2018). Previous work in our laboratory has proved that it has an obvious hypolipidemic effect, but its mechanism is not clear yet.

Intestinal flora can play a key role in the body's physiological regulation, such as the host's material metabolism to protect the host, and playing a role in the immunity, anti‐tumor and improving the liver function of the host (Hu et al., 2017). Intestinal flora can determine the occurrence and progression of metabolic disorders by affecting the host's energy metabolism and immune system. The pathogenesis of hyperlipidemia may be closely related to intestinal flora since an imbalance of intestinal microecological flora is often found in hyperlipidemia patients, and the imbalance can conversely aggravate lipid metabolism disorder, leading to a vicious circle (Lv et al., 2018). It has been found that polysaccharides can improve the integrity of intestinal barrier function, reduce the intestinal mucosal damage, promote the growth and reproduction of beneficial bacteria related to intestinal hyperlipidemia, and inhibit the growth and reproduction of harmful bacteria to regulate and maintain their normal physiological activities by shaping the intestinal flora (Du et al., 2013; El Kaoutari et al., 2013; El‐Hindawy, 2022). The products from polysaccharides degraded by intestinal flora, including lactic acid, acetic acid, propionic acid, and butyric acid, can regulate the pH and microbial diversity in intestines, and provide energy for the body, playing a key role in the protection of the normal peristalsis and barrier of intestines (El‐Hindawy, 2022; Okeke et al., 2014; Uebanso et al., 2017). Further study on whether IOP can exert its hypolipidemic activity by regulating intestinal flora is needed.

The acidic *I. obliquus* polysaccharide (IOP‐A) was extracted and separated, a high‐fat diet was given to rats to establish a rat hyperlipidemia model for observing the hypolipidemic effect of the IOP‐A, and the relationship between its hypolipidemic effect and the regulation of intestinal flora was investigated based on the regulation of intestinal flora to reveal the hypolipidemic mechanism in this study, which was expected to provide such a scientific foundation for the further implementation of functional foods and drugs related to *I. obliquus*.

## 2 MATERIALS AND REAGENTS

### 2.1 Materials

Specific Pathogen Free‐grade male SD rats (4–5 weeks old and 190.0 ± 2.0 g) were provided by Changchun Yisi Experimental Animal Technology Co., Ltd. With animal license SCXK (Ji)‐2016–0003. An ordinary feed (Rat pellet feed) and a high‐fat feed were purchased from Jilin Medical University, and the formula of high‐fat feed is shown in Table 1.

**TABLE 1:** Formula of high‐fat feed

Ingredient	%
Sodium cholate	0.2
Calcium hydrophosphate	0.6
Cholesterol	2
Casein	10
Lard	15
Sucrose	20
Basal feed	52.2

All rats were acclimated to the laboratory environment for 7 days before experiments. They could freely eat and drink in an atmosphere with such a humidity and temperature of 50% to 60% at 20.0–22.0°C.

*Inonotus obliquus* was purchased from Suwei Microbial Research Co., Ltd.; TC, TG, HDL‐C, and LDL‐C determination kits were obtained from Jiancheng Bioengineering Institute; Horseradish peroxidase‐labeled goat anti‐rabbit IgG (H + L) was purchased from Abcam Company; Monoclonal antibodies of SREBP‐1C, CYP7A1, and β‐actin were provided by Affinity Biosciences; protein marker was purchased from GE Healthcare Company; AxyPrep PCR Cleanup Kit/35314KB1 was obtained from Axygen Company; Phusion Hot Start Flex 2X Master Mix was purchased from Yitao Biological Instrument Co., Ltd. The other reagents were analytically pure.

### 2.2 Instruments

High‐Performance Liquid Chromatography was purchased from Shimadzu; Tecan Infinite M200 NanoQuant Absorbance Microplate Reader was provided by Tecan; UV‐2550 ultraviolet–visible spectrophotometer was obtained from Shimadzu; Fluorescence/Chemiluminescence Imaging System was purchased from Beijing SageCreation Science Co., Ltd.; Tanon‐2500 gel imager was purchased from Tanon Science & Technology Co., Ltd.; A200 PCR instrument was obtained from Hangzhou LongGene Scientific Instruments Co., Ltd.

## 3 METHODS

### 3.1 Preparation of the IOP‐A

*Inonotus obliquus* was ground into powders at room temperature, and the *I. obliquus* powders were sifted through a 60‐mesh sieve. The sifted *I. obliquus* powders were dried to the constant weight after removing impurities with anhydrous ethanol, then immersed in deionized water overnight at a substance to liquid ratio of 1:20 (w/v), and extracted by heat reflux extraction method at 90°C for 2 h, which was repeated three times (Li, Lu, et al., 2020; Li, Sheng, et al., 2020). The extracts were filtered and mixed, then the mixture was concentrated by vacuum decompression at 60°C, and the concentrated filtrate was added with four times the volume of anhydrous ethanol for the precipitation of the extract overnight. Then, the precipitate was dissolved in distilled water and a dialysis bag with a molecular mass of 3500 Da was used to dialyze the precipitate‐distilled water solution in distilled water for 48 h for removing the small molecular compounds in it, then lyophilized at −80°C for obtaining a crude IOP, and the crude IOP was classified by DEAE ion‐exchange chromatography to obtain the IOP‐A (Li, Lu, et al., 2020; Li, Sheng, et al., 2020).

### 3.2 Analysis of chemical composition and monosaccharide composition of IOP and IOP‐A

Phenol‐sulfuric acid, m‐hydroxybiphenyl and Coomassie brilliant blue methods were used to determine the content of total polysaccharide, uronic acid and protein in IOP and IOP‐A, in which glucose, D‐galacturonic acid and bovine serum albumin were used as the standard substances, respectively.

Two mg of the IOP‐A were dissolved in 1 ml of 2M HCl–methanol solution, hydrolyzed at 80°C for 8 h, and then hydrolyzed for 1 h at 120°C with 1 ml of 2M trifluoroacetic acid. 1‐phenyl‐3‐methyl‐5‐pyrazolone was used to precolumn‐derivatize the hydrolysate, and a Shimadzu High‐Performance Liquid Chromatography system (SPD‐10AVD UV–VIS detector and LC‐10ATvp pump) and DIKMA Inertsil ODS‐3 (4.6 mm × 150 mm) were used to detect it. According to the retention time and peak area of standard monosaccharide, the monosaccharide composition was identified and the content was calculated (Onul et al., 2012).

### 3.3 Establishment of rat hyperlipidemia model and rat administration

Male Sprague Dawley rats were divided into four groups randomly, namely the blank control group (NG), the model group (MG), the lovastatin group (PG), and the acidic *I. obliquus* polysaccharide group (IOP‐A), eight rats in each group. Rats in the NG group received 100 g/kg of the basal diet daily and those in the MG, PG and IOP‐A groups with 100 g/kg of the high‐fat diet as described above daily. At the same time, 8.4 mg/kg of lovastatin was administered to rats in the PG group, 450 mg/kg of the IOP‐A was administered to rats in the IOP‐A group, and an equivalent amount of distilled water was administered to rats in the MG and NG groups once daily by gavage (Li, Lu, et al., 2020; Li, Sheng, et al., 2020). The high‐fat feeding and administration took place for an 8‐week duration. All experiments using experimental animals were reviewed and approved by the Experimental Animal Committee of Beihua University.

### 3.4 Measurement of body weights and organ indexes

During feeding, the body weights of rats were measured once weekly, the liver and spleen of rats were removed and washed with normal saline at the eighth week after the start of the feeding period (Li, Lu, et al., 2020; Li, Sheng, et al., 2020). The liver index and spleen index were calculated according to the following equation after weighing the wet liver and spleen.

$\text{Organ index}\;\left(\%\right)=\text{organ mass}\;\left(\mathrm{mg}\right)/\text{animal weight}\;\left(g\right)\times 100\%$.

### 3.5 Biochemical indicator determination

The rats were anesthetized with urethane, their blood was collected through the abdominal aorta. The blood was separated by centrifugation at 1917 *g* for 15 min to obtain serum, which was then stored at −20°C. The serum levels of HDL‐C, LDL‐C, TC and TG were determined following the kit manufacturers' instructions (Li, Lu, et al., 2020; Li, Sheng, et al., 2020).

### 3.6 Observation on the pathological changes of hepatic tissue

The hepatic tissue was fixed in 10% neutral formalin fix solution for the routine pathological sampling, sectioning and H&E staining (Li, Lu, et al., 2020; Li, Sheng, et al., 2020). Pathological changes in the liver tissue were examined under an optical microscope (Dechesne et al., 2016).

### 3.7 Analysis of intestinal contents and flora by high‐throughput sequencing

Fecal samples of cecal contents of rats were taken under sterile conditions. A DNA extraction kit was used to extract the total DNA of microorganisms in the fecal samples according to the instructions of the kit. 1% Agarose gel electrophoresis was performed to detect the extracted genomic DNA and the extracted DNA was kept at −20°C for use. The V4 region of the 16S rRNA gene of the samples was amplified by PCR and sequenced by Illumina high‐throughput sequencing. The specific primers with the barcode to the V4 region of 16S rRNA gene was synthesized, and TransGen AP221‐02: TransStartFastpfu DNA polymerase reaction system was applied for the PCR amplification, in which each sample was replicated three times. The PCR products in the same sample were mixed, and the mixture was detected by 2% agarose gel electrophoresis. An AxyPrepDNA gel extraction kit was used to recover the PCR products after gel cutting, and then Tris–HCl was used to elute them and 2% agarose gel electrophoresis was conducted to detect them. The PCR products were quantified using a QuantiFluor™‐ST blue fluorescence quantitative system according to the preliminary quantitative results of electrophoresis, and then mixed in the corresponding proportions. In accordance with the overlapping relationship, paired‐end reads obtained by sequencing were firstly spliced, and the sequence quality was controlled and filtered. The Operational Taxonomic Units (OTU) diversity, alpha diversity, beta diversity, and comparison in biological taxonomy were performed for the analysis of the species differences among groups after distinguishing the samples.

### 3.8 Western blotting analysis

An appropriate amount of RIPA protein lysis buffer was transferred into 100 mg of the hepatic tissue, and the solution was homogenized with a homogenizer. After 1 h of lysis on ice, the homogenate was centrifuged (4°C, 10 min) to obtain the supernatant (Li, Lu, et al., 2020; Li, Sheng, et al., 2020). The BCA method was used to draw the standard curve, and the concentration of proteins was determined and adjusted. The SDS‐PAGE electrophoresis on the proteins was performed to separate them, and then they were transferred onto PVDF membranes. Then, the membranes were blocked with 5% skimmed milk powder and shaken at room temperature for 2 h; after washing them, the membranes were incubated with the primary antibody diluents of CYP7A (1: 1:1000), SREBP‐1C (1:1000), and β‐actin (1:20000) overnight; after washing them, the membranes were incubated with the secondary anti‐Rabbit antibody (1:2000) at room temperature for 1 h (Li, Lu, et al., 2020; Li, Sheng, et al., 2020). After washing the membranes, the images were developed and photographed by a gel imager after the membranes were incubated with ECL luminescent liquid.

### 3.9 Statistical analysis

The data from all the groups were measured three times. The data were presented as mean ± standard deviation (x ± *s*). The statistics analysis was performed using IBM SPSS Statistics for Windows, version 20.0 (IBM Corp.). The differences between the control group and the experimental group were evaluated by *t*‐test. *p* < .05 means that there is a difference in the data, with a statistical significance.

## 4 RESULTS

### 4.1 Preparation of the IOP‐A

#### 4.1.1 Extraction of IOP

The dialysis with a 1000 Da dialysate bag was performed to obtain IOP with a yield of 8.4% of the raw material, in which the ash was removed.

#### 4.1.2 Chemical composition and monosaccharide composition of IOP and IOP‐A

The sugar content was 72.7%, the uronic acid content was 1.6%, and the protein content was 5.2% in IOP, as shown in Table 2. IOP was classified by DEAE‐cellulose column ion‐exchange chromatography and eluted with 0.5 M NaCl solution to obtain an acid sugar fraction, named IOP‐A, and its yield was 43.8%, its total sugar content was 73.1%, and its glucuronic acid content was 8.7%, as shown in Table 2. As shown in Table 3, the IOP‐A was mainly composed of Man (13.08%), GlcA (4.42%), Rha (12.01%), GalA (9.93%), Glc (19.26%), Gal (22.29%), Xyl (8.69%), Ara (5.80%), and Fuc (4.52%).

**TABLE 2:** Composition of IOP and IOP‐A

Polysaccharides	Yield (%)	Total sugar (%)	Glucuronic acid (%)	Protein (%)	Ash (%)
IOP	8.4	72.7	1.6	5.2	4.9
IOP‐A	43.8	73.1	8.7	4.7	4.9

**TABLE 3:** Monosaccharide composition of the IOP‐A

Monosaccharide (mol%)	Man	GlcA	Rha	GalA	Glc	Gal	Xyl	Ara	Fuc
IOP‐A	13.08	4.42	12.01	9.93	19.26	22.29	8.69	5.80	4.52

**FIGURE 1:**
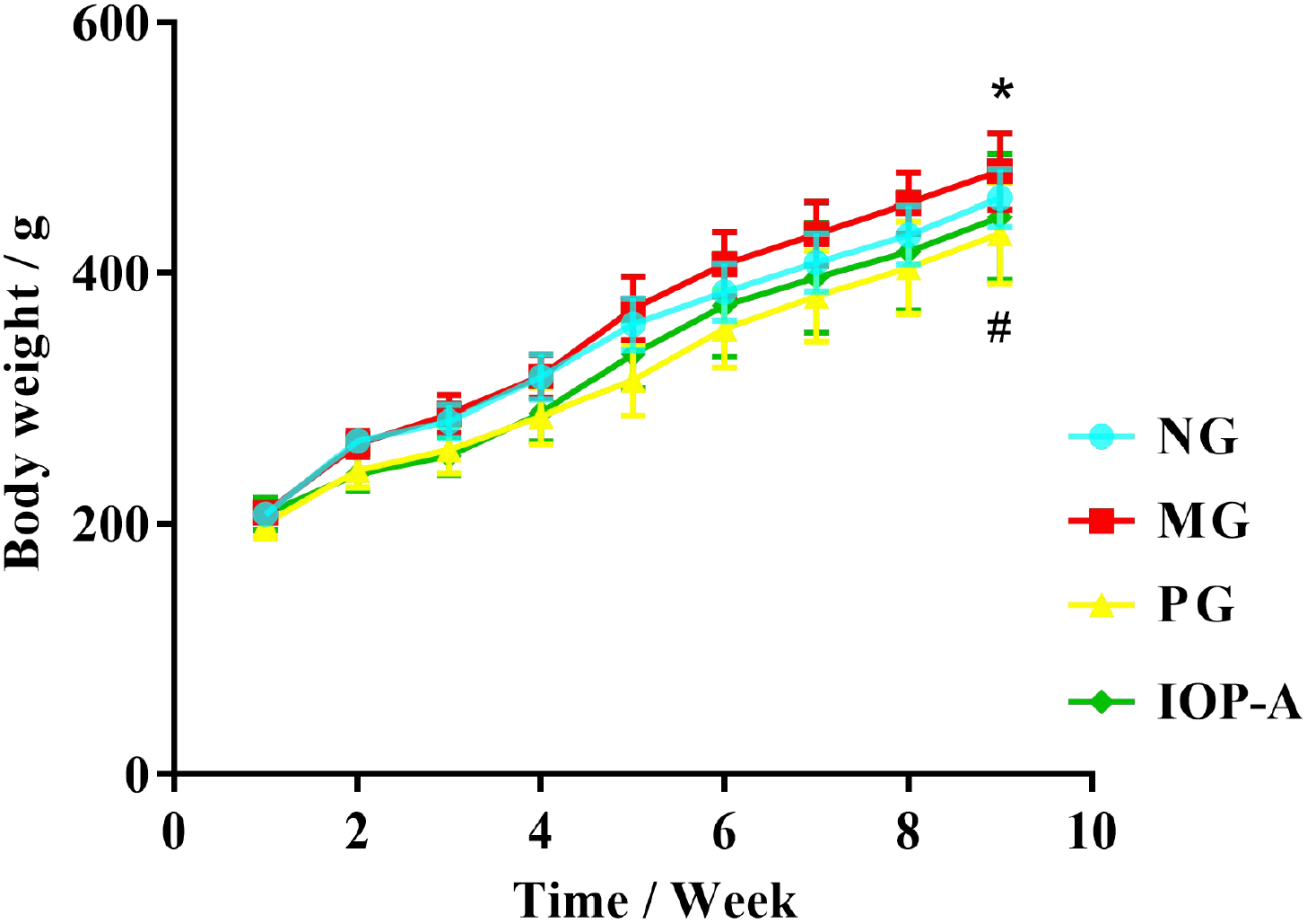
Effect of the IOP‐A on the body weight of rats. *n* = 8; compared with the NG group, *p < .05; compared with the MG group, ^#^ *p* < .05. IOP‐A, acidic *Inonotus obliquus* polysaccharide group; MG, model group; NG, the normal group; PG, positive group

There were no significant differences in body weights between the four groups before the administration of the different agents. The rats' body weights increased continuously in the NG group after the administration, while those in the MG group increased rapidly on the fourth week after the administration and increased significantly compared with those in the NG group on the ninth week (*p* < .05); the increasing trend in the PG group was comparable to that in the IOP‐A group, whereas the body weights in the IOP‐A group were less than those in the MG group, especially significantly on the ninth week (*p* < .05), indicating a slowly increased body weight in the IOP‐A group, as shown in Figure 1.

As shown in Table 4, the liver index or the spleen index of rats in the MG group was considerably higher than that in the NG group, respectively (*p* < .05), while the liver index and the spleen index in the PG group and the IOP‐A group were considerably lower than those in the MG group (*p* < .05).

**TABLE 4:** Effects of the IOP‐A on organ indexes of rats

Parameter	NG	MG	PG	IOP‐A
Liver index (%)	2.470 ± 0.094	2.869 ± 0.198*	2.548 ± 0.128^#^	2.538 ± 0.104^#^
Spleen index (%)	0.153 ± 0.012	0.170 ± 0.015*	0.154 ± 0.015^#^	0.157 ± 0.004^#^

*Note*: Compared with the NG group, *p < .05; compared with the MG group, ^#^ *p* < .05. NG, normal group; MG, model group; PG, positive group; IOP‐A, acidic *Inonotus obliquus* polysaccharide group.

### 4.2 Effects of the IOP‐A on biochemical indicators of rats

As seen in Table 5, compared with those in the NG group, the levels of TC, TG and LDL‐C were considerably increased (*p* < .05), and those of HDL‐C were considerably decreased (*p* < .05) in the serum of rats in the MG group; compared with those in the MG group, the levels of TC, TG and LDL‐C were considerably decreased (*p* < .05), and those of HDL‐C were considerably increased (*p* < .05) in the serum of rats in the PG group, and the levels of TC, TG and LDL‐C were considerably decreased (*p* < .05), and those of HDL‐C were considerably increased (*p* < .05) in the serum of rats in the IOP‐A group.

**TABLE 5:** Effects of the IOP‐A on serum biochemical indicators of rats (mean ± *s*, *n* = 8)

Group	TC (mmol/g)	TG (mmol/g)	LDL‐C (mmol/g)	HDL‐C (mmol/g)
NG	1.365 ± 0.339	1.167 ± 0.244	0.515 ± 0.094	1.161 ± 0.180
MG	2.155 ± 0.351*	2.260 ± 0.533*	0.853 ± 0.211*	0.817 ± 0.203*
PG	1.573 ± 0.247^#^	1.155 ± 0.303^#^	0.519 ± 0.092^#^	1.112 ± 0.181^#^
IOP‐A	1.607 ± 0.296^#^	1.240 ± 0.290^#^	0.551 ± 0.060^#^	1.174 ± 0.267^#^

*Note*: Compared with the NG group, *p < .05; compared with the MG group, ^#^ *p* < .05. NG, normal group; MG, model group; PG, positive group; IOP‐A, acidic *Inonotus obliquus* polysaccharide group.

### 4.3 Effects of the IOP‐A on pathological changes in the hepatic tissue of rats

**FIGURE 2:**
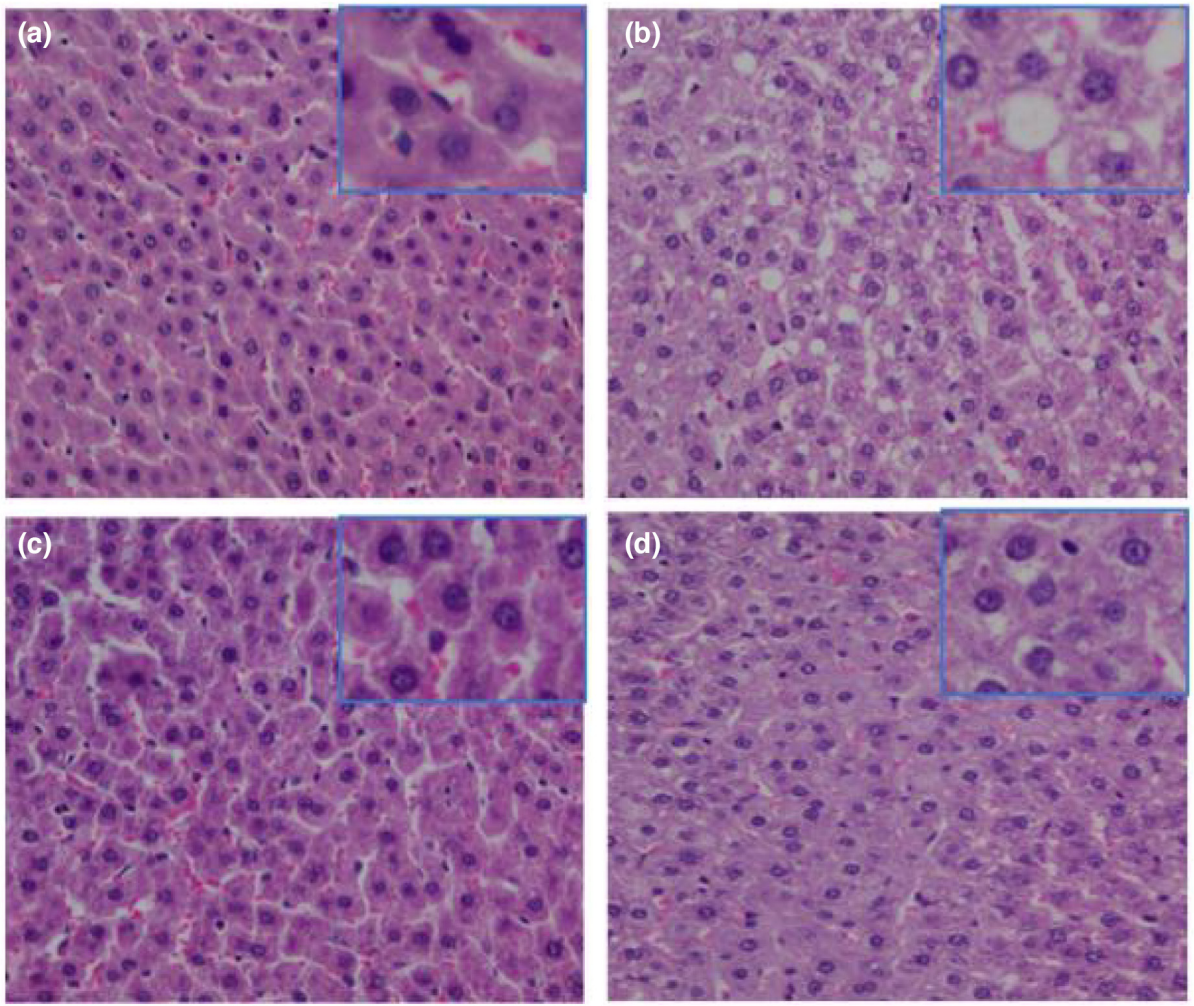
HE staining pathological sections of the liver (4 × 100). (a) the NG group: the normal group; (b) the MG group: the model group; (c) the PG group: the positive group; (d) the IOP‐A group: the acidic *Inonotus obliquus* polysaccharide group.

No pathological abnormality in the hepatic tissue of rats was found in the NG group, with a normal hepatocyte size, an orderly arranged hepatic cord, and no fat vacuole and fat infiltration in hepatocytes (Figure 2a). Some pathological changes could be found in the hepatic tissue of rats in the MG group, such as significantly increased steatosis hepatocytes, extremely swollen and edematous hepatocytes, more hepatocytes with a round shape and disordered hepatic cords (Figure 2b). However, in contrast to MG group, fat vacuoles (Figure 2d) were significantly reduced and hepatic cords were more closely arranged in the PG group (Figure 2c) and the IOP‐A group (Figure 2).

### 4.4 Comparison of intestinal flora at OTU level in rats

**FIGURE 3:**
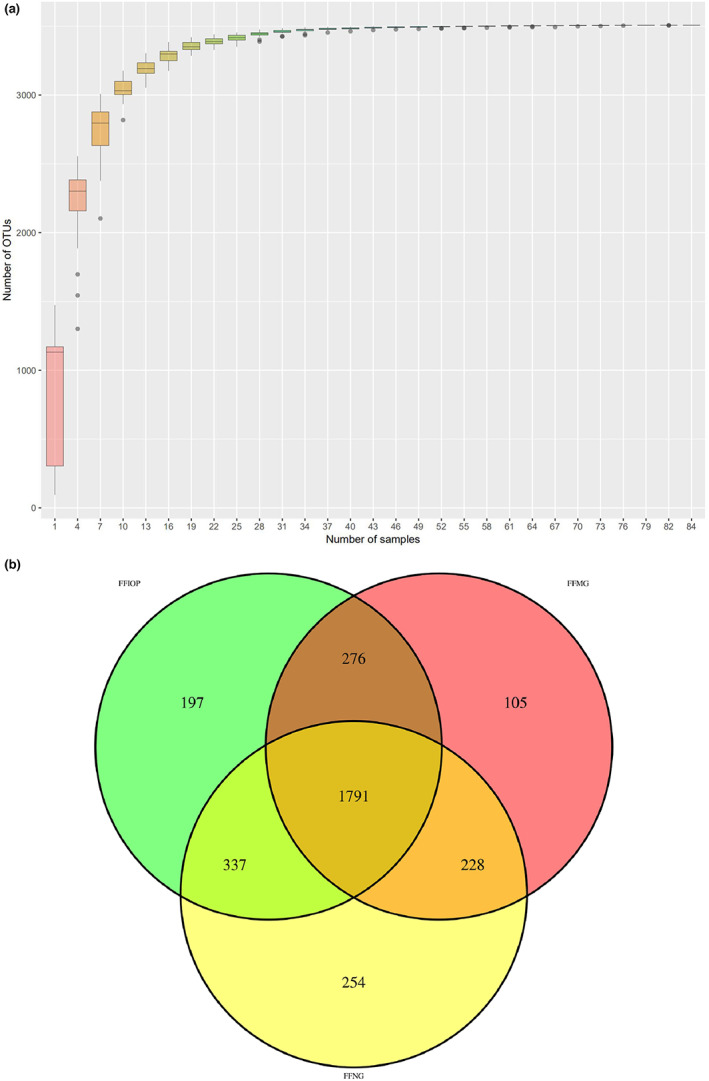
OTU analysis chart. (a) OTU clustering analysis chart; (b) OTU distribution Venn diagram; FFIOP, acidic *Inonotus obliquus* polysaccharide group; FFMG, model group; FFNG, normal group

Differences and similarities among the various samples were statistically analyzed at the OTU level, and the curve tended to be horizontal, suggesting that the sample size of this experiment was sufficient to ensure the accuracy of the results, as shown in Figure 3a. 1791 OTUs were shared in all the samples, and the results showed a species abundance of the NG group > the IOP‐A group > the MG group, as shown in Figure 3b.

### 4.5 Alpha diversity analysis of intestinal flora in rats

**FIGURE 4:**
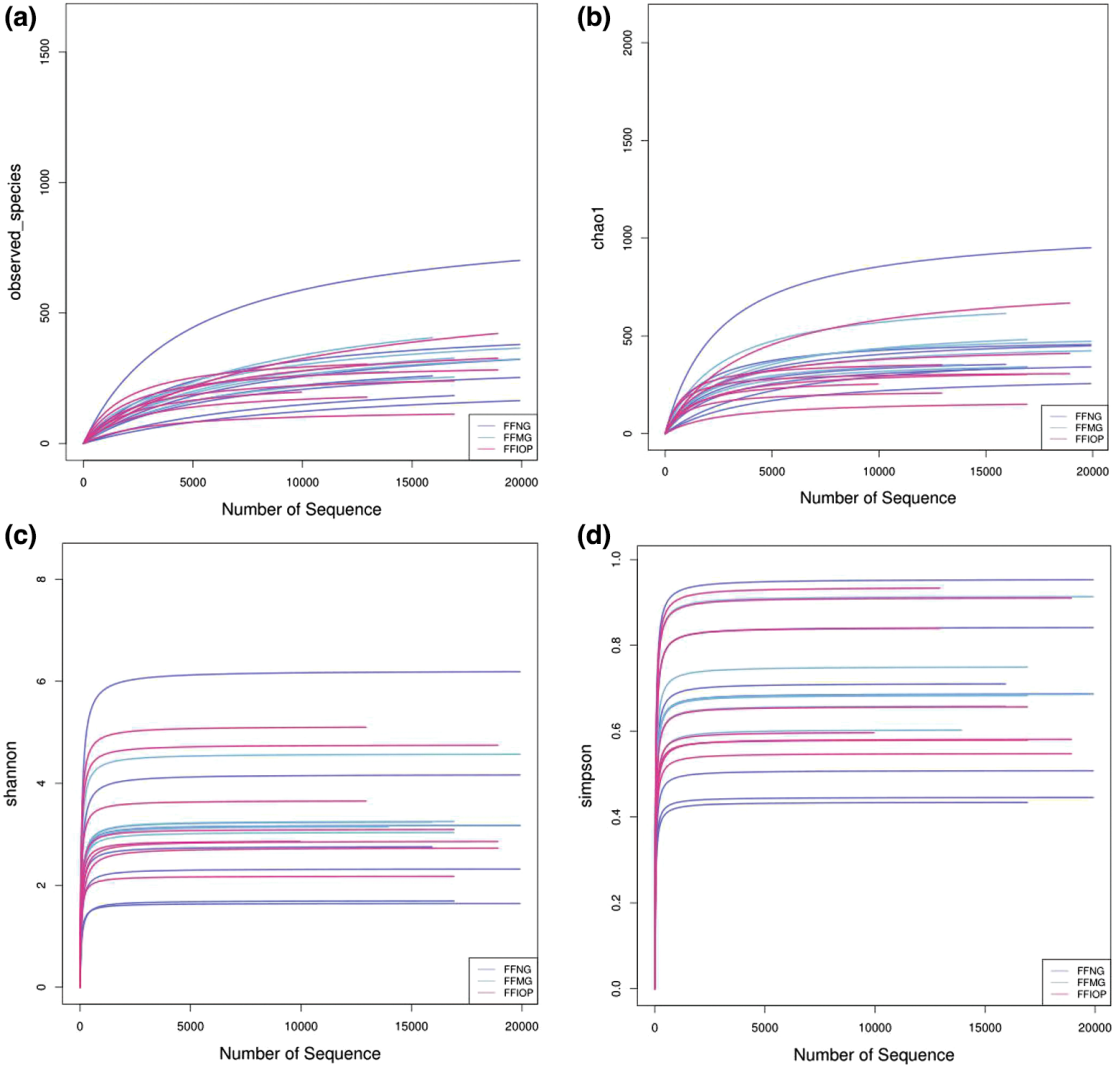
Alpha diversity analysis. (a) Observed_species of FFNG versus FFMG versus FFIOP; (b) Chao1 of FFNG versus FFMG versus FFIOP; (c) Shannon of FFNG versus FFMG versus FFIOP; and (d) Simpson of FFNG versus FFMG versus FFIOP; FFIOP, the acidic *Inonotus obliquus* polysaccharide group; FFMG, model group; FFNG: normal group

The Chao1 index and observed_species indicated the NG group > the IOP‐A group > the MG group in the number of species, and the Simpson and Shannon indexes both indicated that the richness and evenness of the IOP‐A group were higher than those in the MG group, while the diversity of the total bacterial community in the NG group was higher, as shown in Figure 4.

### 4.6 Beta diversity analysis of intestinal flora in rats

**FIGURE 5:**
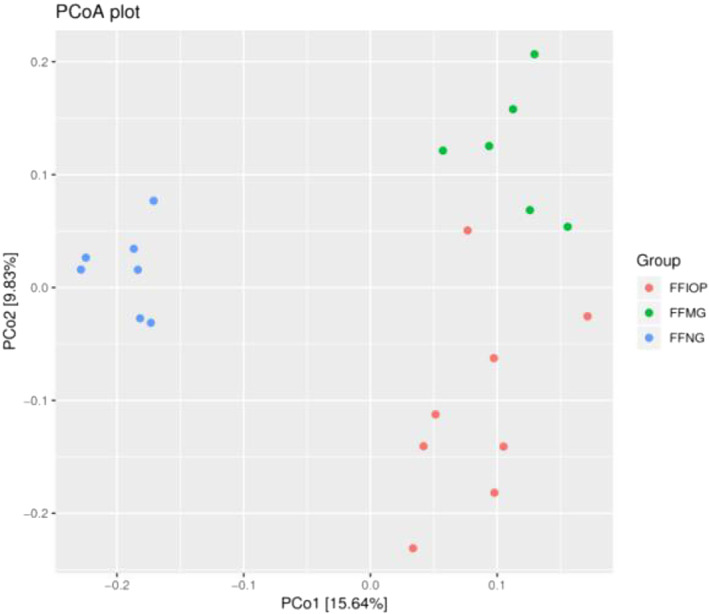
Beta diversity analysis chart. PCoA of FFIOP versus FFMG versus FFNG; FFIOP, the acidic *Inonotus obliquus* polysaccharide group; FFMG, model group; FFNG, normal group

The abscissa indicates the first principal component, and the contribution value of the first principal component to the sample difference was 15.64%, and the ordinate indicates the second principal component and the contribution value of the second principal component to the sample difference was 9.83%; each point in the figure represents a sample, and the three independent circles represent the NG group, the MG group, and the IOP‐A group, respectively, in which the correlation between the MG and the IOP‐A groups were stronger, as shown in Figure 5.

### 4.7 Comparison of the intestinal flora in taxonomy in rats

#### 4.7.1 Comparison of the intestinal flora in rats at the phylum level

**FIGURE 6:**
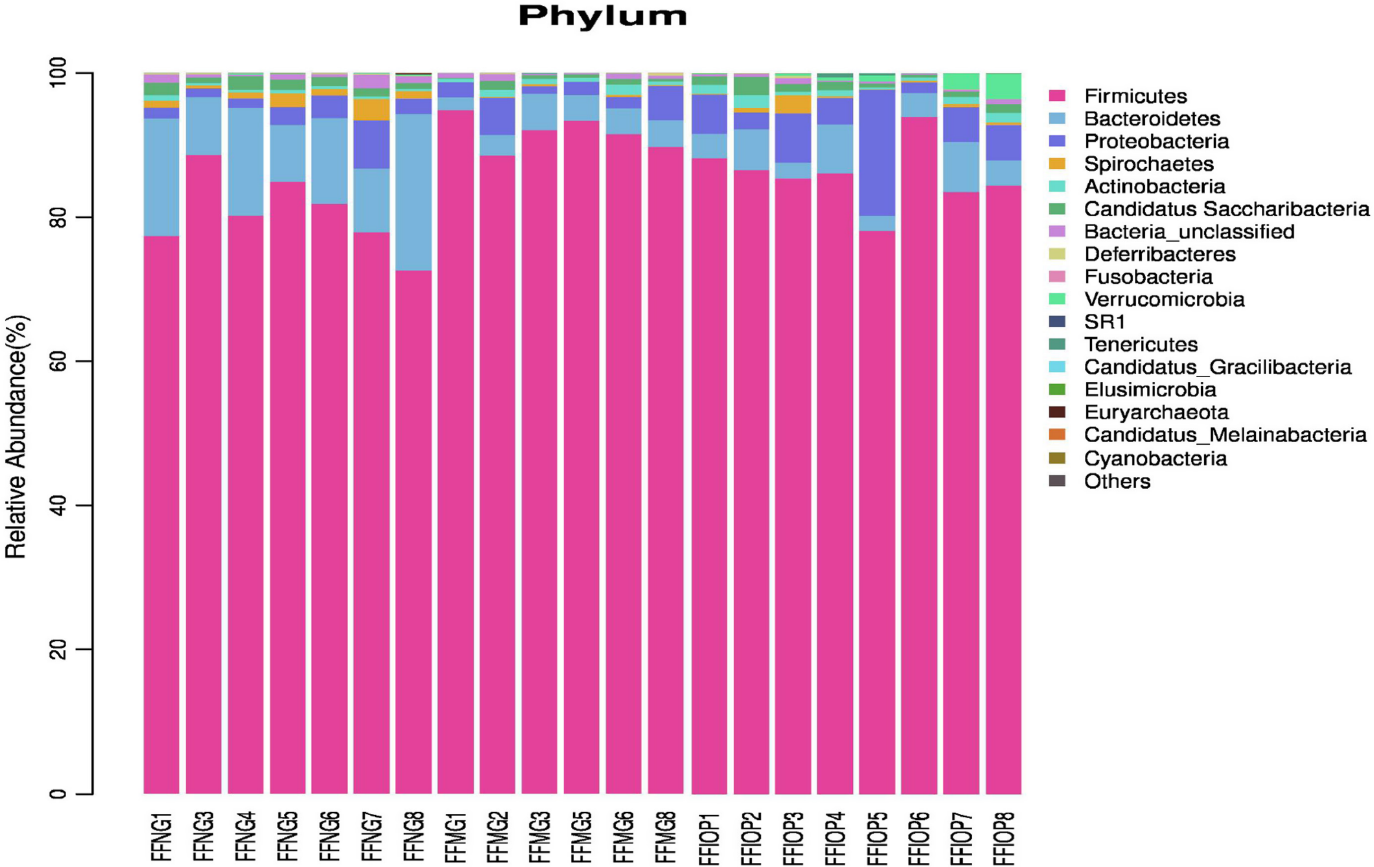
Histogram of fecal microflora at the phylum level. The IOP‐A regulate the composition of intestinal flora by increasing the relative abundance of Firmicutes and reducing the relative abundance of Bacteroides. FFIOP, acidic *I. obliquus* polysaccharide group; FFMG, model group; FFNG: normal group

A total of 17 bacterial communities were identified at the phylum level of rectal fecal microorganisms in the NG group, the MG group, and the IOP‐A group (Figure 6), including *Firmicutes*, *Bacteroidetes*, *Proteobacteria*, *Actinobacteria*, *Spirocheetes*, *Candidatus saccharibacteria*, *Bacteria‐unclassified*, *Deferrobacteria*, *Fusobacteria*, *SR1*, *Tenericutes*, *Verrucomicrobia, Candidatus Gracilibacteria*, *Elusimicrobia*, *Euryarchaeota*, *Candidatus_Melainabacteria* and *Cyanobacteria*.

The difference analysis of specific bacteria among the groups at the phylum level showed that the abundance of *Bacteroidetes* and *Firmicutes* was higher in the NG, MG, and IOP‐A groups, accounting for 80.47%, 91.67%, and 82.75% respectively, and the proportions were 12.83%, 3.43%, and 5.48%, respectively (Figure 6).

Compared with that in the NG group, *Verrucomicrobia* was down‐regulated (*p* < .01) and *Firmicutes* was up‐regulated (*p* < .05) in the MG group. Compared with that in the MG group, *Verrucomicrobia* was up‐regulated *(p* < .01*)*, *Bacteroidetes* was up‐regulated (*p* < .05) and *Firmicutes* was down‐regulated (*p* < .05) in the IOP‐A group, while the proportion of *Firmicutes*/*Bacteroidetes* was decreased from 26.72 to 15.10 after the administration of the IOP‐A (*p* < .05) (Figure 6).

#### 4.7.2 Comparison of the intestinal flora in rats at the genus level

**FIGURE 7:**
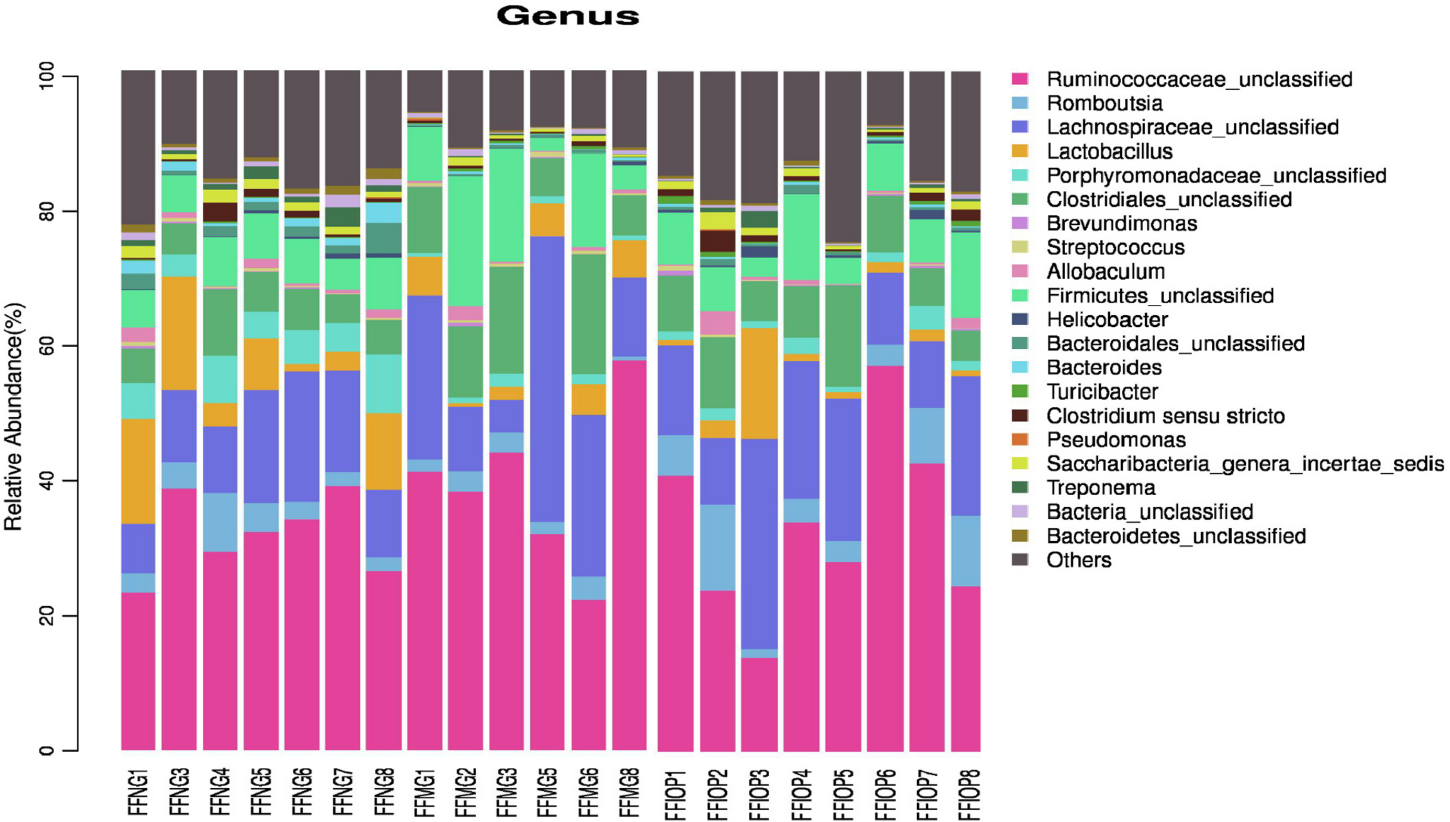
Histogram of fecal microflora at the genus level. The IOP‐A modulates lipid metabolism by up‐regulating Bacteroidales_unclassified and inhibiting Streptococcus in hyperlipidemic rats. FFIOP, acidic *Inonotus obliquus* polysaccharide group; FFMG, model group; FFNG: normal group

A total of 20 bacterial communities were identified at the genus level in the rectal fecal microorganisms of the NG, MG, and IOP‐A groups (Figure 7), including 20 bacterial communities, *Ruminococcaceae_unclassified*, *Romboutsia*, *Lachnospiraceae_unclassified*, *Lactobacillus*, *Porphyromonadaceae_unclassified*, *Clostridiales_unclassified*, *Brevundimonas*, *Streptococcus*, *Allobaculum*, *Firmicutes_unclassified*, *Helicobacter*, *Bacteroidales_unclassified*, *Bacteroides*, *Turicibacter*, *Clostridium_sensu_stricto*, *Pseudomonas*, *Saccharibacteria_genera_incertae_sedis*, *Treponema*, *Bacteria_unclassified*, and *Bacteroidetes_unclassified*.

The difference analysis of specific bacterial genus among the groups at the genus level indicated that the richness of *Ruminococcaceae_unclassified*, *Lachnospiraceae_unclassified* and *Lactobacillus* was higher in FFNG, FFMG, and FFIOP groups, in which *ruminococcaceae_unclassified* accounted for 31.67%, 38.94% and 32.83%, and the proportion of *lachnospiraceae_unclassified* was 12.62%, 19.34% and 16.98%, and that of *Lactobacillus* was 8.37%, 3.27% and 4.82%, respectively (Figure 7).

The difference analysis of specific bacterial communities among the groups at the genus level indicated that compared with that in the NG group, the richness of *Bacteroidales_unclassified* was considerably down‐regulated (*p* < .01) and that of *streptococcus* was significantly up‐regulated (*p* < .05) in the MG group, and compared with that in the MG group, the richness of *Bacteroidales_unclassified* was considerably up‐regulated (*p* < .05) and that of *Streptococcus* was considerably down‐regulated (*p* < .05) in the IOP‐A group (Figure 7).

### 4.8 Effects of the IOP‐A on CYP7A1 and SREBP‐1C protein expressions in the hepatic tissue of rats

**FIGURE 8:**
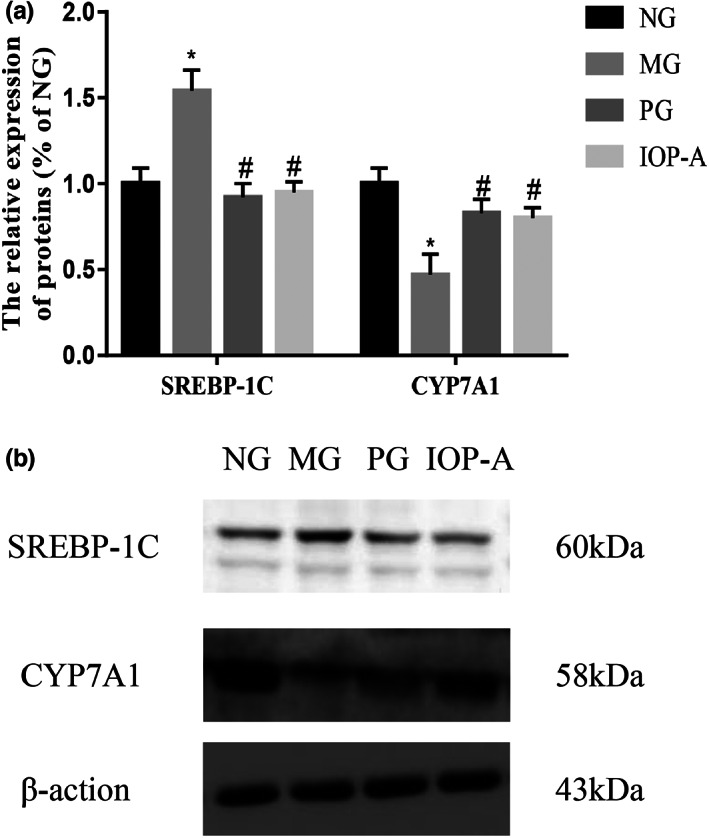
Effects of the IOP‐A on CYP7A1 and SREBP‐1C protein expressions in the hepatic tissue of rats. (a) Relative expressions of SREBP‐1C and CYP7A1 protein in the hepatic tissue of rats; (b) Electrophoretic map of SREBP‐1C and CYP7A1 expressions in the hepatic tissue of rats. *n* = 8; Compared with the NG group, *p < .05; compared with the MG group, ^#^ *p* < .05. IOP‐A, acidic *Inonotus obliquus* polysaccharide group; MG, the model group; NG, the normal group; PG, positive group

The expression of SREBP‐1C protein in the MG group was considerably higher than that in the NG group (*p* < .05), while the expression of CYP7A1 protein in the MG group were considerably lower than that in the NG group (*p* < .05); the expression of SREBP‐1C protein in the PG and IOP‐A groups were considerably lower than that in the MG group (*p* < .05), while the expression of CYP7A1 protein in the PG and IOP‐A groups were considerably higher than that in the MG group (*p* < .05; Figure 8).

## 5 DISCUSSION

Hyperlipidemia is a metabolic disorder syndrome characterized by abnormal increase of lipid constitutes in plasma, and has been recognized as one of the main causes of fatty liver now (Chang et al., 2015). The liver is considered to be a vital organ for the uptake of fat, the metabolism of *free fatty acid*, and synthesis and secretion of cholesterol, phospholipid and lipoproteins, and when the fat in the blood exceeds the metabolic capacity of the liver, fat will be deposited in hepatocytes, leading to the degeneration of hepatocytes, and eventually fatty liver (van Driel et al., 2016; Zhang et al., 2016). Hepatomegaly can be induced by the intake of a high‐fat diet for long term, and an increased liver index and an increased spleen index may indicate hyperlipidemia to a certain extent (Cho et al., 2017; Junior et al., 2016). A rat hyperlipidemia model was induced by a high‐fat diet in this study, and the liver index and spleen index increased significantly, the number of steatosis hepatocytes increased significantly and the arrangement of hepatic cords were disordered, and of TC, TG and LDL‐C levels in the serum increased significantly, while the HDL‐C level decreased significantly in the hyperlipidemia rats (Li, Lu, et al., 2020; Li, Sheng, et al., 2020). After the administration of the IOP‐A, the liver index and spleen index were decreased, the fatty liver was relieved, the serum TC, TG and LDL‐C levels were significantly lower, while the serum HDL‐C level was significantly higher, indicating that the IOP‐A can effectively regulate lipid metabolism in hyperlipidemia rats.

7 Alpha‐hydroxylase (CYP7A1), mainly distributed in the liver, is the key enzyme of TC metabolism and the rate‐limiting enzyme at the first step of TC metabolism to bile acid in the liver, and it can catalyze the decomposition of TC into bile acid to play a key role in maintaining lipid metabolism homeostasis (Chu et al., 2013; Lee et al., 2018). One of the key transcription factors of TG metabolism, SREBP‐1C, can regulate fat and fatty acid synthesis, playing a key role in the lipid toxicity mechanism (Hasegawa et al., 2019; Wang et al., 2018). After the administration of the IOP‐A, the CYP7A1 expression in the hepatic tissue increased and the expression of SREBP‐1C protein decreased significantly, indicating that the IOP‐A may promote the metabolism of cholesterol by activating CYP7A1 and inhibiting SREBP‐1C in hyperlipidemia rats.

Intestinal microflora is considered to be involved in lipid metabolism, and the intestinal absorption is the basis for Chinese medicines to exert their effects, so intestinal flora is likely to be a new target for regulating lipid metabolism disorders (Fadrosh et al., 2014). The high‐fat diet‐induced hyperlipidemia will destroy the balance of intestinal flora, leading to lipid metabolism disorders, and then more serious hyperlipidemia, which may form a vicious cycle (Li et al., 2018). It was found in our study that there were differences in the number of OTUs among the groups, and the analysis of alpha diversity and beta diversity showed that there were differences in the richness of intestinal flora, that is, the lower species richness in the MG group and the significantly higher species richness in the IOP‐A group, indicating that the balance of intestinal microflora could be destroyed by the high‐fat diet, and the IOP‐A could make the intestinal flora form a relatively stable community structure. Studies have found that the Firmicutes (F) to Bacteroidetes (B) ratio in the intestinal microflora of obese mice can increase significantly (Abdallah Ismail et al., 2011; Xue et al., 2016), while the value of F/B decreases after the level of blood lipids is reduced (Bortolin et al., 2018; Yin et al., 2015). As reported, fungal polysaccharides inhibit Firmicutes to a large extent, thereby tending to balance Bacteroidetes, improve the expression of dominant bacteria in the intestinal tract of rats, and regulate the composition of the intestinal flora (Li, Lu, et al., 2020; Li, Sheng, et al., 2020). Our study revealed that the number of Firmicutes and Bacteroidetes was higher in the NG, MG, and IOP‐A groups. Following the administration of the IOP‐A to hyperlipidemia rats, the relative abundance of Firmicutes decreased, and the proportion of Firmicutes/Bacteroidetes decreased. Therefore, the IOP‐A may affect the composition of intestinal flora by reducing the relative abundance of Firmicutes and increasing the relative abundance of Bacteroidetes. At the same time, after the administration of the IOP‐A in hyperlipidemia rats, Bacteroidales_unclassified was up‐regulated and Streptococcus was down‐regulated, which were considered as the flora closely related to hyperlipidemia. Bacteroidales_unclassified belongs to butyrate‐producing bacteria and a high‐fat diet can induce the decrease of intestinal flora to lead to the decrease of butyrate, and the decrease of butyrate will destroy the balance of pH value in the living environment of intestinal flora and butyrate can promote cholesterol transport by increasing the mRNA level and the secretion of phospholipid transporters (Chen et al., 2014; Tang et al., 2017). Streptococcus can increase the production of endotoxin to aggravate the inflammatory reaction, leading to dyslipidemia (Guo et al., 2011; Tarrah et al., 2018). These results suggest that the IOP‐A can regulate lipid metabolism mainly by up‐regulating Bacteroidales_unclassified, down‐regulating the harmful bacterium Streptococcus in hyperlipidemia rats.

Bile acids produced by gut microbes affect hyperlipidemia (Duan et al., 2021). Bile acids, the major metabolites of cholesterol in the liver, are formed by the CYP7A1 enzyme and enhance the absorption of fats, nutrients, and lipophilic vitamins as well as regulate lipid, glucose, and energy metabolism. Aside from that, gut microbes are capable of degrading carbohydrates into monosaccharides and converting them into hydrogen, carbon dioxide, methane, and short‐chain fatty acids, which provide energy for the host (Mahamuni et al., 2012). As a key transcription factor in the metabolism of TG, SREBP‐1C can regulate the synthesis of fat and fatty acids following excessive carbohydrate intake (Tang et al., 2017). The study found that after administration of the IOP‐A to the MG group, the expression of CYP7A1 protein in the hepatic tissue was considerably increased, and the expression of SREBP‐1C protein in the hepatic tissue was considerably decreased. And the relative abundance of Firmicutes and the ratio of Firmicutes to Bacteroidetes decreased in the intestinal microflora. Consequently, the IOP‐A could exert hypolipidemic effects by activating the CYP7A1 enzyme involved in cholesterol metabolism, inhibiting the SREBP‐1C factor in lipid metabolism and regulating the balance of the intestinal microflora structure.

In this study, the hypolipidemic activity of the IOP‐A and its correlation with the regulation of intestinal microflora, and the target of the IOP‐A at the molecular level, were clarified, which may lay a foundation for the study on the action of *I. obliquus* and its mechanism.

## 6 CONCLUSION

The IOP‐A has a hypolipidemic effect, which may be due to its ability to regulate the expression of CYP7A1 and SREBP‐1c, as well as to correct an imbalance in the structure of the intestinal microflora. The study clarifies that IOP‐A regulates the expression of CYP7A1 and SREBP‐1c, and explores part of the mechanism, but it cannot rule out the combined effects of other pathways on blood lipid regulation. The composition of *I. obliquus* is complex, and its anti‐hyperlipidemic mechanism needs further comprehensive and in‐depth research. Consequently, the experiment provides experimental and theoretical support for the further development of functional foods and medicines based on *I. obliquus*.

## Data Availability

The data that support the findings of this study are available from the corresponding author upon reasonable request.
